# Choroidal Osteoma and Secondary Choroidal Neovascularization Treated with Ranibizumab

**DOI:** 10.4274/tjo.86658

**Published:** 2017-08-15

**Authors:** Almila Sarıgül Sezenöz, Sezin Akça Bayar, Gürsel Yılmaz

**Affiliations:** 1 Başkent University Hospital, Ophthalmology Clinic, Ankara, Turkey

**Keywords:** Choroidal osteoma, choroidal neovascularization, intravitreal ranibizumab

## Abstract

A 47-year-old female patient presented with a complaint of decreased vision in the right eye. Her visual acuity was 0.16 in the right eye and 1.0 in the left eye. Fundus examination revealed a slightly elevated, yellowish-white lesion with regular borders at the macula of the right eye. Early and late hyperfluorescence related with choroidal neovascularization (CNV) was detected in the right eye on fundus fluorescein angiography. B-scan ultrasonography revealed a hyperechoic choroidal lesion with acoustic shadowing. The lesion was diagnosed as choroidal osteoma. The patient received 3 injections of intravitreal ranibizumab. After 4 months, the visual acuity of the right eye was 0.9 and the CNV had regressed. Follow-up at about 7 months revealed reduced visual acuity in the right eye with an increase in subretinal fluid. An additional ranibizumab injection was administered. In this case report, we discuss the findings and treatment of a rare case of choroidal osteoma with secondary CNV.

## INTRODUCTION

Choroidal osteoma is a rare ossifying benign tumor with unknown pathogenesis.^1^ It was first identified in 1978 by Gass et al.^2^ The tumor is generally unilateral and located in juxtapapillary and macular region. Although it occurs more frequently in young women, men and middle-aged people may also be affected.^3,4^ Despite being a benign tumor, it may cause serious vision loss due to pigment epithelium atrophy, serous retinal detachment, and most commonly choroidal neovascularization (CNV).^3,5^ While patients usually present with blurred vision, metamorphopsia, photophobia, and vision field defects, 8-30% of patients are asymptomatic.^3,4^ Choroidal osteoma is diagnosed by clinical examination. It appears in fundus examination as a slightly elevated, yellowish-white or orange-colored vascularized lesion with well-defined borders and pigment epithelium changes.^3,4,6^ Fundus autofluorescence, computed tomography, ultrasonography (USG), magnetic resonance imaging, optical coherence tomography (OCT), and fundus fluorescein angiography (FFA) assist diagnosis. Treatment targets complications. Here, we aimed to discuss a patient diagnosed with choroidal osteoma and secondary CNV who was treated with intravitreal ranibizumab.

## CASE REPORT

**Figure 1:**
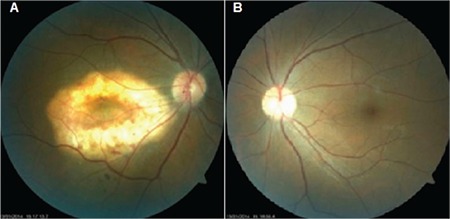
A) Hypopigmented choroidal osteoma in the right macula, B) The left fundus appears normal

**Figure 2:**
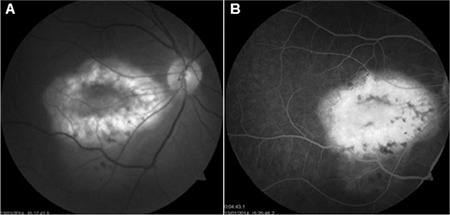
A) Fundus fluorescein angiography shows the osteoma area in the early phase, B) Increased active leakage in the late period in the area compatible with the osteoma

**Figure 3:**
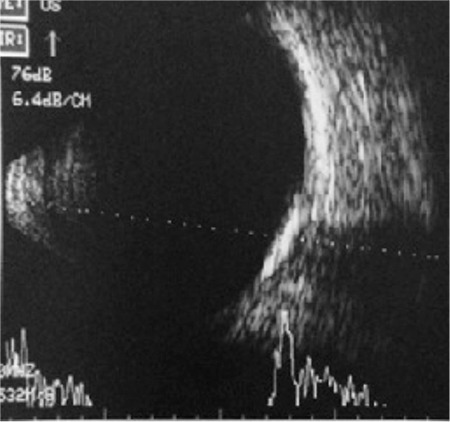
B-scan ultrasonography imaging of the right eye reveals a choroidal lesion causing acoustic shadowing

**Figure 4:**
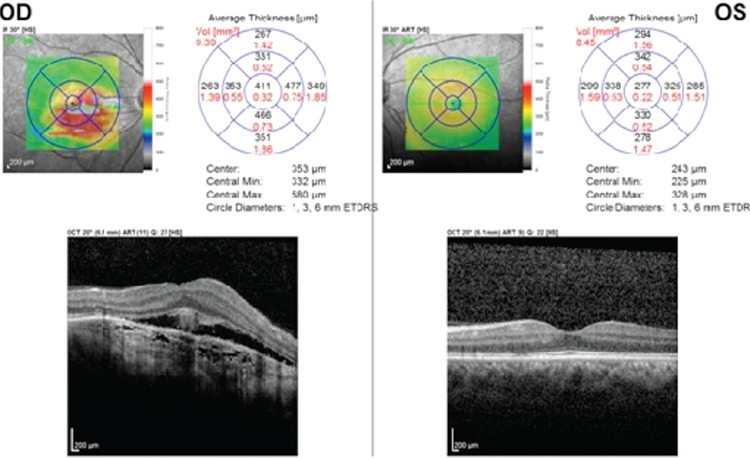
Pre-treatment optical coherence tomography imaging shows subretinal fluid and an area of choroidal neovascularization with a lesion situated in the cornea and optic shadowing behind it

**Figure 5:**
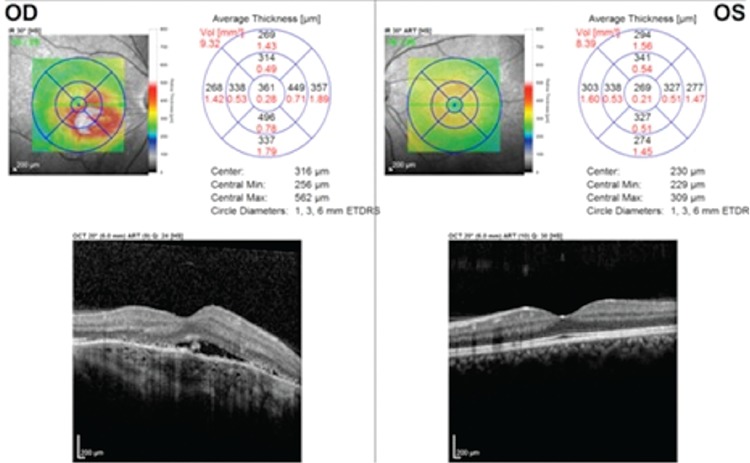
The subretinal fluid is reduced after intravitreal ranibizumab injection

A 47-year-old female patient presented with complaints of decreased vision in her right eye. On examination, her visual acuity was 0.16 in the right eye and 1.0 in the left eye. Slit-lamp anterior segment examination and intraocular pressures were normal in both eyes. Fundus examination revealed a yellowish-white lesion that had well-defined borders and was slightly raised from the surface of the retina located at macula of the right eye, while the left eye was normal (Figure 1A, B). FFA revealed early hyperfluorescence increasing in later stages and CNV in the region compatible with the lesion in the right eye (Figure 2A, B). B-scan USG in the right eye revealed a hyperechoic choroidal lesion causing acoustic shadowing (Figure 3). Spectral domain OCT revealed subretinal fluid in the right eye (Figure 4). Based on those findings, the patient was diagnosed with choroidal osteoma and secondary CNV. The patient was administered 3 intravitreal ranibizumab injections at 1-month intervals. In follow-up examination at 4 months post-injections, visual acuity had improved to 0.9 and OCT imaging showed regression of the subretinal fluid (Figure 5). Although the patient’s vision was stable during that period, a decline in visual acuity was observed 3 months later. An additional intravitreal ranibizumab injection was administered when the visual acuity in the right eye reached 0.4. At final follow-up 2 months after the injection, OCT revealed that the subretinal fluid had regressed, and visual acuity had improved to 0.8. The patient’s condition was stable during the 2-year follow-up period and no additional injections were required.

## DISCUSSION

The main causes of vision loss in choroidal osteoma are the development of CNV, subfoveal fluid, and photoreceptor degeneration.^5^ CNV is seen more frequently among patients exhibiting a combination of hemorrhage and surface irregularities.^5^ Shields et al.^3^ observed CNV development in 31% of their 61 choroidal osteoma patients, while Aylward et al.^4^ reported the incidence as 47% in their study of 36 patients. Although the mechanism by which CNV develops is not well understood, researchers have suggested that retinal pigment epithelium damage CNV in the underlying choroid, or that osteoma itself has neovascular membrane extensions.^1,7^ FFA and OCT are useful methods for identifying CNV.

There is no standard method for the treatment of choroidal osteoma. Patients must be followed regularly and secondary complications must be treated as appropriate. In the management of CNV secondary to choroidal osteoma, partial success has been achieved using thermal laser photocoagulation and photodynamic therapy (PDT) for extrafoveal lesions, and transpupillary thermotherapy and PDT for subfoveal lesions. However, some studies showed that these treatment methods might leading to tumor decalcification, thus increasing retinal damage.^8,9,10^

It is believed that laser photocoagulation may not have adequate efficacy in cases of CNV secondary to choroidal osteoma due to insufficient tumor melanine and a thinned, degenerated RPE-Bruch’s membrane complex.^1^ While PDT provides short-term improvement in visual acuity, it has been demonstrated that retreatment may be necessary and final visual acuity may decline.^11^ Surgical removal of subfoveal CNV membranes results in favorable anatomic outcomes, but researchers have reported the procedure unsuccessful in terms of visual acuity.^12^

Another method employed in the treatment of these patients is intravitreal anti-vascular endothelial growth factor (anti-VEGF). Bevacizumab and ranibizumab injections have been tested for this purpose, and positive outcomes were reported in both anatomy and visual acuity.^5,6^ It is thought that in choroidal osteoma cases, normal tissues are also damaged during the process, and VEGF expression is increased as a result of choroidal and retinal ischemic stress and chronic inflammation. RPE damage together with thinning of Bruch’s membrane and the choriocapillaris may contribute to the development of CNV. Thus, increased VEGF supports abnormal neovascularization. Therefore, anti-VEGF agents may be effective in treatment.^13^

The first trials of intravitreal bevacizumab in the literature were conducted for this purpose and yielded favorable results.^6,13^ In a case reported by Ahmadieh and Vafi,^6^ visual acuity improved from 20/200 to 20/20 after intravitreal bevacizumab injection and was preserved at this level throughout a 9-month follow-up period. In another case study from Kubota-Taniai et al.,^13^ visual acuity improved to 0.7 from 0.2 after bevacizumab injection and was preserved over the course of 4 years of follow-up. The highly effective responses obtained with anti-VEGF injections were attributed to enhanced passage of the bevacizumab through the thinned and degenerated RPE and Bruch’s membrane to the subretinal area, thus increasing the drug’s efficacy.^6^

The first reported use of intravitreal ranibizumab injection for CNV secondary to choroidal osteoma was by Song and Roh^5^ in 2009; they found that CNV had regressed and visual acuity had improved from 20/200 to 20/100 at 6 months post-injection. In another case report, Gupta et al.^14^ observed CNV regression after ranibizumab injection and no recurrence was detected in 30 months of follow-up. Wu et al.^15^ reported a case in which visual acuity improved from 20/800 to 20/30 after 3 injection and no recurrence was observed during 1.2 years of follow-up. Mansour et al.^16^ demonstrated in their series consisting of 26 cases that intravitreal ranibizumab and bevacizumab were effective. In our case, visual acuity improved from 0.16 to 0.9 after 3 monthly injections. However, a decrease in visual acuity 3 months after the final injection and CNV recurrence necessitated another ranibizumab injection.

In conclusion, although intravitreal ranibizumab injection is highly beneficial in the treatment of CNV secondary to choroidal osteoma in terms of visual acuity and anatomic recovery, recurrence may be observed, as our case also shows. Therefore, patients should be examined regular at monthly intervals, and treatment should be supported by repeated intravitreal anti-VEGF injections when required.
